# Pneumonia Risk in Institutionalized Older Adults With Severe Functional Dependency: An Exploratory Analysis Using Standardized Long‐Term Care Assessment Data

**DOI:** 10.1111/ggi.70452

**Published:** 2026-03-19

**Authors:** Yuichi Ohteru, Tomoyuki Kakugawa, Keita Murakawa, Masahiro Kakugawa, Tsunahiko Hirano, Kazuto Matsunaga

**Affiliations:** ^1^ Department of Pulmonology and Gerontology Graduate School of Medicine, Yamaguchi University Ube Japan; ^2^ Medical Corporation WADOKAI, Hofu Rehabilitation Hospital Hofu Japan; ^3^ Department of Respiratory Medicine and Infectious Disease Graduate School of Medicine, Yamaguchi University Ube Japan; ^4^ Department of Respiratory Medicine National Hospital Organization Yamaguchi Ube, Medical Center Ube Japan

**Keywords:** cognitive dysfunction, hypoalbuminemia, nursing homes, pneumonia, weight loss

## Abstract

**Aim:**

To identify risk factors for pneumonia among institutionalized older adults with severe functional dependency, using routinely available clinical information from the standardized “Doctor's Written Opinion” for long‐term care insurance.

**Methods:**

This retrospective observational study included 257 institutionalized older adults with severe functional dependency (median age, 88 years; 55 men and 202 women) residing in a Japanese nursing home between January 2014 and May 2020. Risk factors for pneumonia were analyzed using medical records and data from the standardized “Doctor's Written Opinion” for long‐term care insurance.

**Results:**

During a median follow‐up of 2.1 years, 51 residents (19.8%) developed pneumonia, which was associated with lower overall survival (log‐rank test, *p* = 0.001). Among the 102 residents who died during this period, pneumonia was the most common cause of death (37% of deaths). Multivariate Cox regression analysis revealed male sex (hazard ratio [HR] 4.692, 95% confidence interval [CI] 2.352–9.357), serum albumin level (HR 0.368, 95% CI 0.151–0.898), cognitive ability for daily decision‐making (HR 1.626 per one‐point worsening on a four‐point scale, 95% CI 1.082–2.444), and recent weight loss (≥ 3% over the past 6 months) (HR 2.645, 95% CI 1.195–5.858) as significant risk factors.

**Conclusions:**

Male sex, lower serum albumin level, impaired cognitive ability for daily decision‐making, and recent weight loss were associated with an increased risk of pneumonia. Information routinely available in standardized long‐term care assessments may support the identification of individuals at increased risk of pneumonia in resource‐limited long‐term care settings where comprehensive geriatric assessments are not feasible.

## Graphical abstract

A simple, routinely collected long‐term care assessment identified key predictors of pneumonia—male sex, hypoalbuminemia, impaired cognitive ability for daily decision‐making, and weight loss of ≥ 3% over the past 6 months. Tracking short‐term weight loss provides a practical risk marker for institutionalized older adults with severe functional dependency.

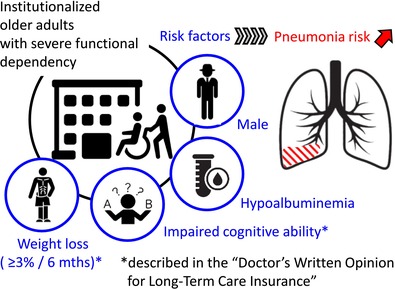

## 1 Introduction

The global aging trend is expected to substantially increase the burden of respiratory infections [1]. Older adults face a higher risk of severe respiratory infections, with pneumonia‐related mortality disproportionately affecting those aged ≥ 65 years [2, 3, 4, 5, 6]. Moreover, community‐acquired pneumonia (CAP) is associated with sustained declines in quality of life (QOL) and activities of daily living (ADL), with many patients failing to regain pre‐disease functional status even 1 year after onset [6, 7, 8]. Pneumonia also imposes a considerable economic burden [9, 10, 11]. These factors underscore the need for effective prevention strategies. Identifying risk factors is essential, particularly through simple, practical tools that can pinpoint high‐risk individuals and enable timely, targeted interventions in resource‐limited settings.

Studies have identified several pneumonia risk factors among older residents of long‐term care facilities. Key individual‐level factors include advanced age [12, 13], male sex [13, 14], cognitive impairment [15, 16, 17, 18], swallowing dysfunction [13, 14], immobility or reduced ADL [12, 13], malnutrition [12, 19], and poor oral hygiene [20, 21, 22, 23, 24, 25, 26]. Evidence also links sarcopenia—characterized by reduced skeletal muscle mass and strength—to lower tongue pressure and respiratory muscle weakness, which increases pneumonia risk in older nursing home residents [27, 28, 29]. These muscle impairments are associated with dysphagia, diminished cough efficacy, and compromised airway protection, the key mechanisms underlying aspiration pneumonia.

Previous studies often relied on complex, time‐consuming assessments requiring specialized equipment, such as videofluoroscopic swallowing studies, respiratory muscle strength testing, and detailed cognitive evaluations (e.g., the Global Deterioration Scale). Similarly, sarcopenia assessment typically involves objective measurements of muscle mass (e.g., bioelectrical impedance analysis or dual‐energy X‐ray absorptiometry), muscle strength (e.g., handgrip dynamometry), and physical performance (e.g., gait speed). These approaches are impractical for routine use in most long‐term care facilities. Despite robust evidence linking these factors to pneumonia risk, studies employing simple, scalable tools suitable for resource‐limited settings are lacking. Addressing this methodological gap is essential for timely prevention in older adults with severe functional dependency residing in institutional care settings.

To address this gap, we used the “Doctor's Written Opinion for Long‐Term Care Insurance” (hereafter referred to as the “doctor's written opinion”)—a standardized and mandatory document in Japan, completed by physicians for all older adults applying for long‐term care services [30, 31]. This form records physical function, cognitive status, nutritional condition, and comorbidities, and determines care levels nationwide. Its simplicity makes it a practical source of information for pneumonia risk assessment among institutionalized older adults with severe functional dependency, especially in resource‐limited settings. We investigated whether information from this document, together with routinely available clinical data, could support the identification of individuals at increased risk of pneumonia without requiring complex clinical assessments.

## 2 Methods

### 2.1 Study Population

This retrospective observational study included all residents admitted to the Hofu Akari‐en Special Nursing Home, a government‐licensed long‐term care facility in Yamaguchi, Japan, between January 2014 and May 2020. In Japan, *Special Nursing Homes* primarily accommodate older adults with substantial physical and/or cognitive impairments who require 24‐h assistance with nearly all ADL. Admission is generally restricted to individuals certified as *Care Level 3–5* under the national long‐term care insurance system, reflecting a high degree of functional dependency and care needs. The facility admission date was defined as the start of the observation period. Residents unable to tolerate oral or enteral feeding were excluded from the study. The study protocol complied with the principles of the Declaration of Helsinki and was approved by the Institutional Review Board of Yamaguchi University Hospital, Ube, Japan (Approval No. 2020‐100). In accordance with the ethical guidelines set by the Japanese Ministry of Health, Labour and Welfare, the requirement for individual informed consent was waived for this retrospective study. Instead, residents' right to refuse participation was communicated via a notice posted on the facility's bulletin board.

### 2.2 Covariates

The characteristics of the study population were obtained from medical records and routinely available clinical data, including age, sex, body mass index (BMI), cognitive function assessed using the Hasegawa Dementia Scale‐Revised (HDS‐R) scores [32], medical history, comorbidities, vaccination status for pneumococcal vaccines (23‐valent pneumococcal polysaccharide vaccine and 13‐valent pneumococcal conjugate vaccine), influenza vaccine, medication history, and laboratory parameters (hemoglobin, albumin, total protein, total cholesterol, and creatinine). Information regarding smoking history could not be obtained because of impaired cognitive abilities or the absence of relevant documentation in medical records. Additional data related to long‐term care insurance included nursing care level, classified into seven categories from *Support Required 1* (indicating high ADL function) to *Care Level 5* (indicating low ADL function); independence degree of daily living for disabled older adults (Table S1); independence degree of daily living for demented older adults (Table S2); impairment of short‐term memory (present or absent); cognitive ability for daily decision‐making (independent, semi‐independent, needing help, or impossible); ability to communicate intentions (possible, a little difficult, only specific requests, or impossible); weight change over the past 6 months (“increase,” “stable,” or “decrease,” with a change exceeding 3% considered clinically significant based on guidelines from the Ministry of Health, Labour and Welfare in Japan); eating ability (independent or requiring complete assistance); and social interaction (independent, requiring partial assistance, or requiring complete assistance). The Charlson Comorbidity Index [33] was calculated based on medical history and comorbidities. The first pneumonia event, including the date of diagnosis and associated outcomes during the observation period, was recorded for each patient.

### 2.3 Clinical and Outcome Assessments

The primary outcome was the time to the first episode of pneumonia during the observation period. Residents were followed from the date of facility admission until the occurrence of the first pneumonia event, hospital admission for any condition other than pneumonia, or loss to follow‐up by May 31, 2020, whichever occurred first. Hospital admission for conditions other than pneumonia and loss to follow‐up were treated as censoring events in the time‐to‐pneumonia analysis.

For survival analysis, residents were followed from the date of facility admission until death from any cause. Residents were censored if they were alive as of May 31, 2020, or lost to follow‐up.

### 2.4 Statistical Analysis

Baseline characteristics—including age, sex, BMI, HDS‐R score, medical history, complications, vaccination history, medication history, laboratory data, and information related to long‐term care insurance—were compared between the pneumonia onset and non‐onset groups. Summary statistics are presented as frequencies for categorical variables or medians and IQR for quantitative variables. Inter‐group differences were evaluated using Fisher's exact test for categorical variables and the Wilcoxon rank‐sum test for quantitative variables. Time to the first episode of pneumonia was assessed using Kaplan–Meier analysis, and pneumonia‐free survival curves were estimated. Differences between Kaplan–Meier curves were assessed using the log‐rank test. For multivariate analysis of pneumonia risk, Cox's proportional hazards regression model was used to explore multiple factors associated with pneumonia development. Adjusted hazard ratio (HR) and 95% confidence intervals (95% CI) were calculated for each risk factor. To ensure reproducibility, no more than five explanatory variables (each with > 50 cases) were included owing to the limited sample size (*N* = 257). Hence, in the multivariate Cox regression modeling, a stepwise selection method with a threshold *p*‐value of 0.05 was performed. The goodness‐of‐fit of the Cox regression model was evaluated using the concordance index (C‐index). The log‐rank test was used to evaluate differences in Kaplan–Meier curves for pneumonia risk factors identified by Cox analysis. In this analysis, numerical variables were treated as binary variables using cutoff values estimated by receiver operating characteristic (ROC) analysis.

All statistical analyses were performed using IBM SPSS Statistics for Windows version 27 (IBM, Armonk, NY, USA).

## 3 Results

### 3.1 Residents' Characteristics

Table S3 presents the baseline characteristics of the nursing home residents included in this study. The study cohort comprised 257 residents (55 men and 202 women) with a median age of 88.0 years (IQR, 84.0–91.5). The median follow‐up duration was 2.1 years (IQR, 1.0–3.7); the median survival time (MST) was 2.7 years from the start of observation.

Overall, 89.5% of residents had a “nursing care level” rating of *Care Level 3*–*5*, and 82.1% had an “independence degree of daily living for disabled older adults” rating of B–C. Based on these functional assessments, > 80% of residents were estimated to have an Eastern Cooperative Oncology Group Performance Status of 3 or 4 [34], indicating marked functional impairment and advanced care needs.

### 3.2 Pneumonia Incidence

**FIGURE 1:**
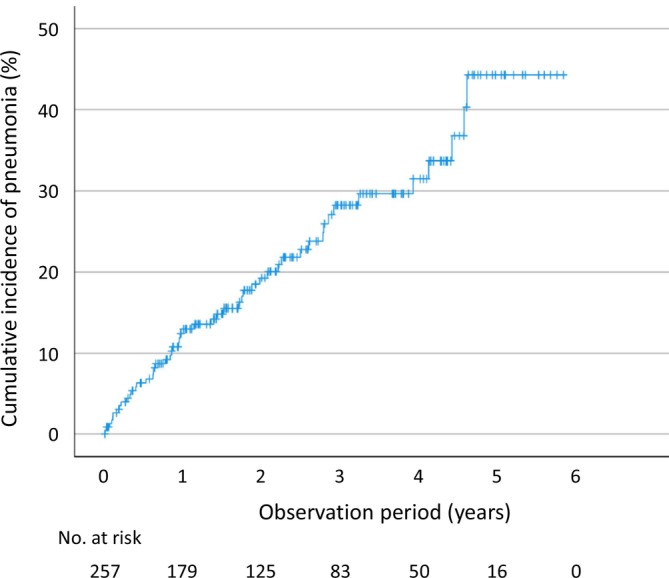
Cumulative incidence of pneumonia in nursing home residents. Kaplan–Meier analysis estimated pneumonia incidence rates of 12.4% at 1 year, 19.2% at 2 years, and 28.2% at 3 years.

During follow‐up, pneumonia occurred in 51 residents (19.8%), while 2 residents (0.8%) were lost to follow‐up. Kaplan–Meier analysis estimated pneumonia incidence rates of 12.4% at 1 year, 19.2% at 2 years, and 28.2% at 3 years (Figure 1).

### 3.3 Impact of Pneumonia on Overall Survival

**FIGURE 2:**
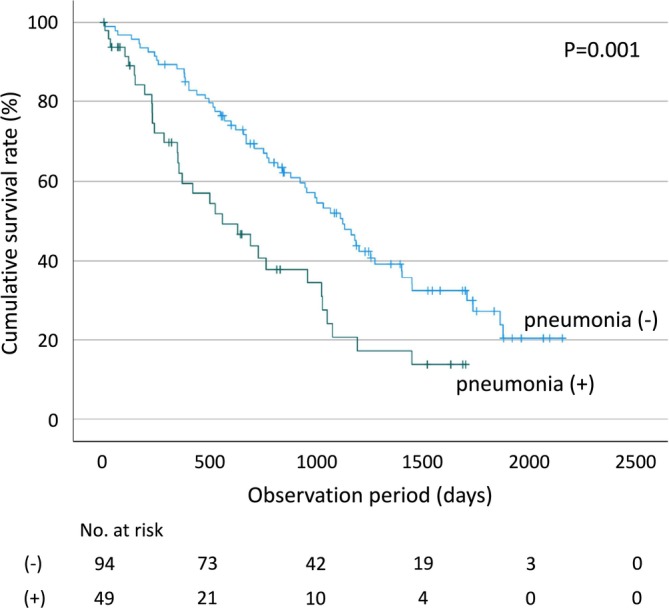
Comparison of cumulative survival rates in pneumonia and non‐pneumonia groups. Nursing home residents who developed pneumonia during the observation period exhibited a significantly shorter median survival time (MST, 558 days) compared with those who did not (1120 days) (log‐rank test, *p* = 0.001).

Pneumonia was significantly associated with overall survival among nursing home residents. Those who developed pneumonia had a significantly shorter MST (558 days) compared with those who did not (1120 days) (log‐rank test, *p* = 0.001) (Figure 2). During the follow‐up period, 102 residents died. According to recorded causes of death, pneumonia was the most common cause, accounting for 37% of all deaths (Figure S1).

### 3.4 Comparison of Baseline Characteristics Between Nursing Home Residents With and Without Pneumonia

Table 1 presents a comparison of the baseline characteristics between residents who developed pneumonia and those who did not. Significant differences were observed between the two groups in sex (*p* = 0.002), HDS‐R score (*p* < 0.001), use of gastric acid‐suppressive drugs (*p* = 0.041), serum albumin levels (*p* = 0.016), cognitive ability for daily decision‐making (*p* = 0.030), ability to communicate intentions (*p* = 0.008), eating ability (*p* = 0.024), and social interaction (*p* = 0.008).

**TABLE 1:** Comparison of baseline characteristics between nursing home residents with and without pneumonia.

	Non‐pneumonia		*n*	Pneumonia		*n*	*p* ^a^
	(*N* = 206)			(*N* = 51)			
Basic information							
Age, median (IQR), years	88.0	(84.0–91.0)	206	88.0	(81.0–92.0)	51	0.807
Sex, *n* (%)			206			51	
Male	36	(17.5)		19	(37.3)		0.002
Female	170	(82.5)		32	(62.7)		
BMI, median (IQR), kg/m^2^	19.7	(17.9–21.5)	205	19.9	(17.9–22.2)	51	0.748
Comorbidities							
HDS‐R, median (IQR)	8.0	(2.5–17.0)	129	1.0	(0.0–4.5)	28	< 0.001
Charlson Comorbidity Index, median (IQR)	2.0	(1.0–3.0)	202	2.0	(1.0–3.0)	51	0.688
Cardiovascular disease, *n* (%)			206			51	
Yes	31	(15.0)		7	(13.7)		0.812
No	175	(85.0)		44	(86.3)		
Cerebrovascular disease, *n* (%)			206			51	
Yes	96	(46.6)		17	(33.3)		0.087
No	110	(53.4)		34	(66.7)		
Chronic lung disease, *n* (%)			204			51	
Yes	5	(2.4)		1	(2.0)		0.659
No	201	(97.6)		50	(98.0)		
Hepatic disease, *n* (%)			203			51	
Yes	12	(5.9)		4	(7.8)		0.536
No	191	(94.1)		47	(92.2)		
Diabetes mellitus, *n* (%)			206			51	
Yes	38	(18.4)		9	(17.6)		0.895
No	168	(81.6)		42	(82.4)		
Solid tumor, *n* (%)			206			51	
Yes	21	(10.2)		6	(11.8)		0.743
No	185	(89.8)		45	(88.2)		
Vaccinations							
Pneumococcal vaccine, *n* (%)			173			39	
Yes	88	(50.9)		17	(43.6)		0.412
No	85	(49.1)		22	(56.4)		
Influenza vaccine, *n* (%)			201			14	
Yes	198	(97.6)		14	(100.0)		0.727
No	3	(2.4)		0	(0.0)		
Medications							
ACE inhibitors, *n* (%)			206			51	
Yes	8	(3.9)		2	(3.9)		0.624
No	198	(96.1)		49	(96.1)		
Gastric acid‐suppressive drugs, *n* (%)			206			51	
Proton pump inhibitor	58	(28.2)		9	(17.6)		0.041
Histamine H2‐receptor antagonists	67	(32.5)		12	(23.5)		
No	81	(39.3)		30	(58.8)		
Anticholinergic agents, *n* (%)			206			51	
Yes	6	(2.9)		1	(2.0)		0.580
No	200	(97.1)		50	(98.0)		
Steroids, *n* (%)			206			51	
Yes	7	(3.4)		1	(2.0)		0.506
No	199	(96.6)		50	(98.0)		
Hypnotics, *n* (%)			206			51	
Yes	55	(26.7)		11	(21.6)		0.453
No	151	(73.3)		40	(78.4)		
Antipsychotics, *n* (%)			206			51	
Yes	53	(25.7)		19	(37.3)		0.101
No	153	(74.3)		32	(62.7)		
Laboratory data							
Serum hemoglobin, median (IQR), g/dL	11.6	(10.6–13.0)	201	11.5	(10.1–12.4)	51	0.172
Serum albumin, median (IQR), g/dL	3.7	(3.4–3.9)	197	3.6	(3.2–3.7)	50	0.016
Serum total protein, median (IQR), g/dL	6.6	(6.3–7.0)	193	6.7	(6.3–7.2)	51	0.391
Serum total cholesterol, median (IQR), mg/dL	172.0	(150.0–196.0)	190	161.0	(138.0–184.0)	50	0.131
eGFR, median (IQR), mL/min/1.73 m^2^	62.1	(47.6–78.4)	202	67.5	(51.3–80.5)	51	0.436
Doctor's written opinion for long‐term care insurance							
Nursing care level, *n* (%)			131			21	
Support Required 1 (High ADL)	0	(0.0)		0	(0.0)		0.353
Support Required 2	0	(0.0)		0	(0.0)		
Care Level 1	9	(6.9)		0	(0.0)		
Care Level 2	7	(5.3)		0	(0.0)		
Care Level 3	29	(22.1)		3	(14.3)		
Care Level 4	63	(48.1)		11	(52.4)		
Care Level 5 (Low ADL)	23	(17.6)		7	(33.3)		
Independence degree of daily living for disabled older adults, *n* (%)^b^			206			51	
Independent	0	(0.0)		0	(0.0)		0.614
J1 (High ADL)	0	(0.0)		0	(0.0)		
J2	2	(1.0)		0	(0.0)		
A1	8	(3.9)		5	(9.8)		
A2	24	(11.7)		7	(13.7)		
B1	33	(16.0)		5	(9.8)		
B2	95	(46.1)		23	(45.1)		
C1	30	(14.6)		7	(13.7)		
C2 (Low ADL)	14	(6.8)		4	(7.8)		
Independence degree of daily living for demented older adults, *n* (%)^c^			206			51	
Independent	6	(2.9)		1	(2.0)		0.102
I (High ADL)	18	(8.7)		0	(0.0)		
IIa	15	(7.3)		3	(5.9)		
IIb	31	(15.0)		6	(11.8)		
IIIa	86	(41.7)		25	(49.0)		
IIIb	26	(12.6)		5	(9.8)		
IV	23	(11.2)		9	(17.6)		
M (Low ADL)	1	(0.5)		2	(3.9)		
Impairment of short‐term memory, *n* (%)			205			50	
Yes	183	(89.3)		47	(94.0)		0.235
No	22	(10.7)		3	(6.0)		
Cognitive ability for daily decision‐making, *n* (%)			205			51	
Independent	15	(7.3)		2	(3.9)		0.030
Semi‐independent	52	(25.4)		7	(13.7)		
Needing help	77	(37.6)		16	(31.4)		
Impossible	61	(29.8)		26	(51.0)		
Ability to communicate intentions, *n* (%)			204			51	
Possible	33	(16.2)		3	(5.9)		0.008
A little difficult	54	(26.5)		6	(11.8)		
Only specific requests	78	(38.2)		25	(49.0)		
Impossible	39	(19.1)		17	(33.3)		
Weight change over the past 6 months (≥ 3% change), *n* (%)			169			37	
Increase or stable	144	(85.2)		28	(75.7)		0.157
Decrease	25	(14.8)		9	(24.3)		
Eating ability, *n* (%)			205			51	
Independent	160	(78.0)		32	(62.7)		0.024
Complete assistance	45	(22.0)		19	(37.3)		
Social interaction, *n* (%)			204			51	
Independent	31	(15.2)		3	(5.9)		0.008
Partial assistance	117	(57.4)		23	(45.1)		
Complete assistance	56	(27.5)		25	(49.0)		

Abbreviations: ACE inhibitor, angiotensin‐converting enzyme inhibitor; ADL, activities of daily living; BMI, body mass index; eGFR, estimated glomerular filtration rate; HDS‐R, Hasegawa Dementia Rating Scale‐Revised; IQR, interquartile range.

^a^ Between‐group differences were evaluated using Fisher's exact test for categorical variables and Wilcoxon's rank‐sum test for quantitative variables.

^b^ Details for the independence degree of daily living for disabled older adults are shown in Table S1.

^c^ Details for the independence degree of daily living for demented older adults are shown in Table S2.

### 3.5 Risk Factors for Pneumonia

**FIGURE 3:**
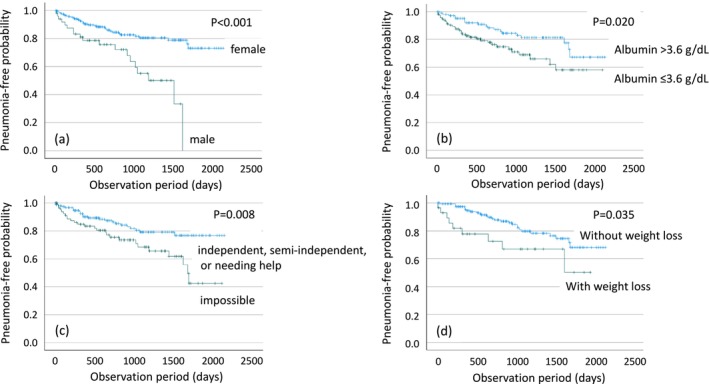
Associations between risk factors for pneumonia and pneumonia‐free probability. Kaplan–Meier curves illustrate pneumonia‐free probability based on the following risk factors: (a) sex, comparing males and females; (b) serum albumin levels, categorized as > 3.6 g/dL and ≤ 3.6 g/dL; (c) cognitive ability for daily decision‐making, divided into two groups: “impossible” and “independent, semi‐independent, or needing help”; and (d) weight loss of ≥ 3% over the past 6 months, categorized into groups with and without such weight loss.

In the univariate Cox analysis (Table 2), male sex, no gastric acid‐suppressive drug use, lower serum albumin level, lower serum total cholesterol level, higher nursing care level, impaired cognitive ability for daily decision‐making, and weight loss of ≥ 3% over the past 6 months were associated with an increased risk of pneumonia. For the multivariate Cox analysis, five risk factors for pneumonia were selected using stepwise selection: sex, use of gastric acid‐suppressive drugs, serum albumin level, cognitive ability for daily decision‐making level, and weight loss of ≥ 3% over the past 6 months. In the final multivariate model, male sex, lower serum albumin levels, impaired cognitive ability for daily decision‐making, and weight loss of ≥ 3% over the past 6 months were independently associated with pneumonia development, with respective HR [95% CI], 4.692 [2.352–9.357], 0.368 [0.151–0.898], 1.626 [1.082–2.444], and 2.645 [1.195–5.858] (Table 2). Here, the HR for “cognitive ability for daily decision‐making” indicates that with each one‐level decline in evaluation (e.g., from independent to semi‐independent), the risk of pneumonia increases by a factor of 1.626. The overall goodness‐of‐fit of the regression model, calculated using the C‐index, was 0.724, indicating moderate discriminative ability of the model. The associations between these four variables and pneumonia‐free probability, as evaluated by the log‐rank test, are shown in Figure 3. ROC curve analysis revealed 3.6 g/dL as a cutoff level for serum albumin in relation to pneumonia risk. The log‐rank test demonstrated that male sex, serum albumin levels ≤ 3.6 g/dL, impaired cognitive ability for daily decision‐making, and weight loss of ≥ 3% over the past 6 months were significantly associated with decreased pneumonia‐free survival probability (*p* < 0.001, *p* = 0.020, *p* = 0.008, and *p* = 0.035, respectively).

**TABLE 2:** Cox analysis for risk factors for pneumonia in nursing home residents.

	Univariate Cox analysis		Multivariate Cox analysis	
	HR (95% CI)	*p*	HR (95% CI)	*p*
Basic information				
Age (years)	1.004 (0.965–1.043)	0.856		
Sex, male	3.004 (1.682–5.365)	< 0.001	4.692 (2.352–9.357)	< 0.001
Body mass index	0.962 (0.877–1.055)	0.412		
Medications				
ACE inhibitors, yes	0.457 (0.110–1.898)	0.281		
Gastric acid‐suppressive drugs, yes	0.567 (0.324–0.991)	0.046	0.612 (0.295–1.270)	0.188
Laboratory data				
Serum albumin level (g/dL)	0.305 (0.154–0.604)	< 0.001	0.368 (0.151–0.898)	0.028
Serum total cholesterol level (mg/dL)	0.991 (0.983–1.000)	0.047		
Doctor's written opinion for long‐term care insurance				
Nursing care level^a^	2.063 (1.169–3.642)	0.013		
Impairment of short‐term memory, yes	1.676 (0.521–5.390)	0.386		
Cognitive ability for daily decision‐making level^b^	1.597 (1.147–2.226)	0.006	1.626 (1.082–2.444)	0.019
Body weight loss of ≥ 3% over the past 6 months, yes	2.239 (1.056–4.748)	0.036	2.645 (1.195–5.858)	0.016

Abbreviations: 95% CI, 95% confidence interval; ACE inhibitor, angiotensin‐converting enzyme inhibitor; HR, hazard ratio.

^a^ Nursing care level was classified into seven categories, from Support Required 1 (indicating high ADL function) to Care Level 5 (indicating low ADL function).

^b^ Cognitive ability for daily decision‐making was classified into four categories: independent, semi‐independent, needing help, and impossible.

## 4 Discussion

Older adults with severe functional dependency are at high risk of pneumonia, which is associated with adverse outcomes [2, 3, 4, 5, 6, 7, 8, 19, 22, 35]. In our cohort, nearly one‐fifth of residents developed pneumonia, which was the leading recorded cause of death (37% of fatalities). Those with pneumonia had a markedly shorter MST compared with those who did not. These findings indicate that pneumonia is not only a frequent complication but is also associated with reduced survival in this population. Identifying high‐risk individuals using feasible, time‐efficient methods is therefore critical. In this study, male sex, lower serum albumin levels, impaired cognitive ability for daily decision‐making, and ≥ 3% weight loss over the past 6 months were independently associated with the occurrence of pneumonia among institutionalized older adults with severe functional dependency.

Importantly, these risk factors can be assessed using simple measures routinely available in long‐term care settings. Cognitive ability for daily decision‐making was rated on a four‐point scale during standard physician–patient interactions, avoiding complex tools such as the Global Deterioration Scale [18, 36, 37, 38, 39]. Similarly, weight loss can be identified through brief patient interviews or routine care documentation, and serum albumin levels are readily available from standard blood tests. Together, these findings may inform a pragmatic, context‐specific approach to pneumonia risk stratification among institutionalized older adults with severe functional dependency, particularly in resource‐limited long‐term care settings.

Sarcopenia is associated with an increased risk of pneumonia in older adults [27, 28, 29, 40, 41]. Weakness of the respiratory and swallowing muscles may reduce cough efficacy and airway protection, thereby predisposing individuals to aspiration [28, 29, 42, 43]. Low BMI, particularly in the underweight range, has been consistently associated with sarcopenia and is frequently regarded as an indirect marker of nutritional and functional vulnerability in older adults [44, 45, 46, 47]. Low BMI is independently associated with an increased risk of pneumonia and pneumonia‐related mortality [17, 48]. Beyond static BMI, prior studies have highlighted the prognostic relevance of weight change itself. Both low body weight and recent weight loss are independent risk factors for CAP in community‐dwelling older adults [49], and substantial changes in body weight over 5 years have been associated with higher pneumonia‐related mortality in a large Japanese cohort [50].

Our findings extend previous observations to a highly vulnerable population—very old institutionalized residents with severe functional dependency—by demonstrating that even modest short‐term weight loss (≥ 3% over 6 months) was associated with a higher likelihood of pneumonia than a persistently low but stable BMI. This finding suggests that tracking trajectories of body weight, rather than relying solely on static BMI values, may provide a dynamic and clinically meaningful marker for pneumonia risk in geriatric care settings. In our cohort, both the pneumonia and non‐pneumonia groups exhibited low median BMI values (19.9 and 19.7, respectively), indicating chronic undernutrition. In this context, further declines in BMI may reflect loss of lean body mass, particularly skeletal muscle, rather than reductions in adiposity. Therefore, unlike community‐dwelling older adults with more heterogeneous body composition, serial declines in BMI among institutionalized older adults with severe functional dependency may serve as a practical surrogate marker of progressive muscle loss, including sarcopenia‐related changes. Prospective studies incorporating direct assessments of muscle mass and function are warranted to validate these observations and to further elucidate the biological pathways linking muscle loss and pneumonia risk in institutionalized older populations.

Malnutrition is a risk factor for pneumonia. In our study, hypoalbuminemia and ≥ 3% weight loss over 6 months were independently associated with pneumonia occurrence among institutionalized older adults with severe functional dependency. Malnutrition increases infection susceptibility through multiple immune impairments [51, 52, 53, 54, 55, 56, 57]. Severe nutritional deficits may also lead to thymic atrophy and attenuated vaccine‐induced antibody production [54]. Taken together, our findings suggest that weight loss and hypoalbuminemia reflect overlapping domains of nutritional and physiological vulnerability that may contribute to increased susceptibility to pneumonia.

Progressive weight loss may also reflect involvement in a vicious cycle: malnutrition increases the risk of dysphagia, which, in turn, reduces dietary intake and worsens nutritional status [58, 59, 60, 61]. Videofluorographic studies have identified malnutrition as an independent risk factor for high‐risk dysphagia [59], while older adults with dysphagia are nearly five times more likely to be undernourished [61]. Poor oral hygiene may further accelerate this cycle [60]. For individuals with ongoing weight loss, tailored nutritional and supportive interventions—such as texture‐modified diets, high‐calorie supplements, and appropriate dental care—may help interrupt this cycle and potentially limit further decline, although prospective interventional studies are needed to confirm their effectiveness.

Hypoalbuminemia, while often considered a marker of malnutrition, also reflects chronic inflammation, which suppresses hepatic albumin synthesis and accelerates protein catabolism [62, 63]. In older adults with CAP, inflammation—rather than nutritional deficits—has been reported to be a major contributor to hypoalbuminemia [64]. Chronic inflammation promotes immunosuppressive pathways and may increase susceptibility to infection [65]. Hypoalbuminemia is associated with diminished humoral immune responses to coronavirus disease 2019 mRNA vaccination in older adults, highlighting its broader relevance to immune dysfunction [66]. In the present study, lower serum albumin level was independently associated with pneumonia occurrence, consistent with earlier reports [67, 68]. Taken together, these findings suggest the importance of disentangling the relative contributions of malnutrition and inflammation. Further investigation is warranted into whether strategies targeting inflammation, in addition to nutritional support, may potentially reduce pneumonia risk among institutionalized older adults with severe functional dependency.

This study has certain limitations. First, the relatively small sample size may limit the generalizability of the findings. Second, the retrospective design introduces potential selection and information biases that cannot be fully excluded. Third, the study was conducted at a single institution, potentially restricting the external validity. In addition, the use of standardized long‐term care assessment data embedded within the Japanese national long‐term care insurance system further limits the generalizability of our findings to broader or international contexts, as these instruments are not routinely used outside Japan. Fourth, although aspiration pneumonia was likely the predominant subtype given the very old age and severe functional dependency of our study population [69, 70, 71, 72], definitive classification was not feasible owing to the lack of widely accepted diagnostic criteria and limited clinical data. Therefore, all pneumonia events were analyzed as a single outcome. Fifth, the observation period was relatively short, possibly limiting the identification of long‐term risk factors. Finally, our analysis focused on the time to the first pneumonia event after admission to provide a uniform baseline and minimize confounding from prior episodes, given that a history of pneumonia is a strong predictor of recurrence [73, 74]. While this approach is methodologically appropriate for the present exploratory study design and limited sample size, it does not capture the full cumulative burden of pneumonia over time. Future multicenter, prospective studies with longer follow‐up in diverse long‐term care systems beyond Japan are warranted to validate these findings and evaluate their broader applicability before any practical application can be considered.

## 5 Conclusion

Male sex, lower serum albumin levels, impaired cognitive ability for daily decision‐making, and weight loss of ≥ 3% over the past 6 months were associated with an increased risk of pneumonia among institutionalized older adults with severe functional dependency. Information routinely available in standardized long‐term care assessments may support the identification of individuals at increased risk of pneumonia in resource‐limited long‐term care settings where comprehensive geriatric assessments are not feasible. In addition, our findings regarding their associations with pneumonia may provide hypothesis‐generating evidence for future prevention research in long‐term care settings.

## Author Contributions

T.K. conceived the fundamental study concept and outlined the methodology. Y.O. and T.K. designed this study. Y.O. and T.K. accessed, verified, and reviewed the data. Y.O. and T.K. wrote the manuscript. All authors had full access to all the data in this study and vouched for the accuracy and completeness of the data, data analyses, and the interpretation and fidelity of the protocol. All authors have reviewed, edited, and approved the final version of the manuscript.

## Funding

This research received no specific grant from any funding agency in the public, commercial, or not‐for‐profit sectors.

## Ethics Statement

The study protocol was approved by the Institutional Review Board of Yamaguchi University Hospital, Ube, Japan (Approval No. 2020‐100).

## Consent

The requirement for individual informed consent was waived for this retrospective study. Resident's right to refuse participation was communicated via a notice posted on the facility's bulletin board.

## Conflicts of Interest

T.K. is an employee of the Department of Pulmonology and Gerontology, Graduate School of Medicine, Yamaguchi University, Ube, Japan, and is funded by the Medical Corporation WADOKAI. Y.O., T.K., and M.K. are employees of Hofu Rehabilitation Hospital, Hofu, Japan, which belongs to the Medical Corporation WADOKAI. M.K. is a board member of Medical Corporation WADOKAI (unpaid). The authors declare no conflicts of interest.

## Supporting information


**Figure S1:** Cause of death among nursing home residents.
**Table S1:** Independence degree of daily living for disabled older adults.
**Table S2:** Independence degree of daily living for demented older adults.
**Table S3:** Baseline characteristics of nursing home residents.

## Data Availability

The data are available to approved individuals upon reasonable request from Yamaguchi University after fulfilling specific requirements.
