# Supplementary material for: Pneumonia Risk in Institutionalized Older Adults With Severe Functional Dependency: An Exploratory Analysis Using Standardized Long‐Term Care Assessment Data
Source: Geriatr Gerontol Int. 2026 Mar 19;26(3):e70452. doi: 10.1111/ggi.70452 (PMC13000870; doi:10.1111/ggi.70452)
Supplement: Supplementary file 1 — Figure S1: Cause of death among nursing home residents. Table S1: Independence degree of daily living for disabled older adults. Table S2: Independence degree of daily living for demented older adults. Table S3: Baseline characteristics of nursing home residents. [file GGI-26-0-s001.docx]

**Online Supplement**

**Title:**

Pneumonia risk in institutionalized older adults with severe functional dependency: An exploratory analysis using standardized long-term care assessment data

**Authors:**

Yuichi Ohteru, Tomoyuki Kakugawa, Keita Murakawa, Masahiro Kakugawa, Tsunahiko Hirano, Kazuto Matsunaga

Table of Contents

Figure S1: Cause of death among nursing home residents …………………………………………………………………………………………………3

Table S1: Independence degree of daily living for disabled older adults …………………………………………………………………………………………………4

Table S2: Independence degree of daily living for demented older adults ...………………………...………………………………………………………………5

Table S3: Baseline characteristics of nursing home residents …………………………………6

**Supplementary Figures**

Figure S1: Causes of death among nursing home residents


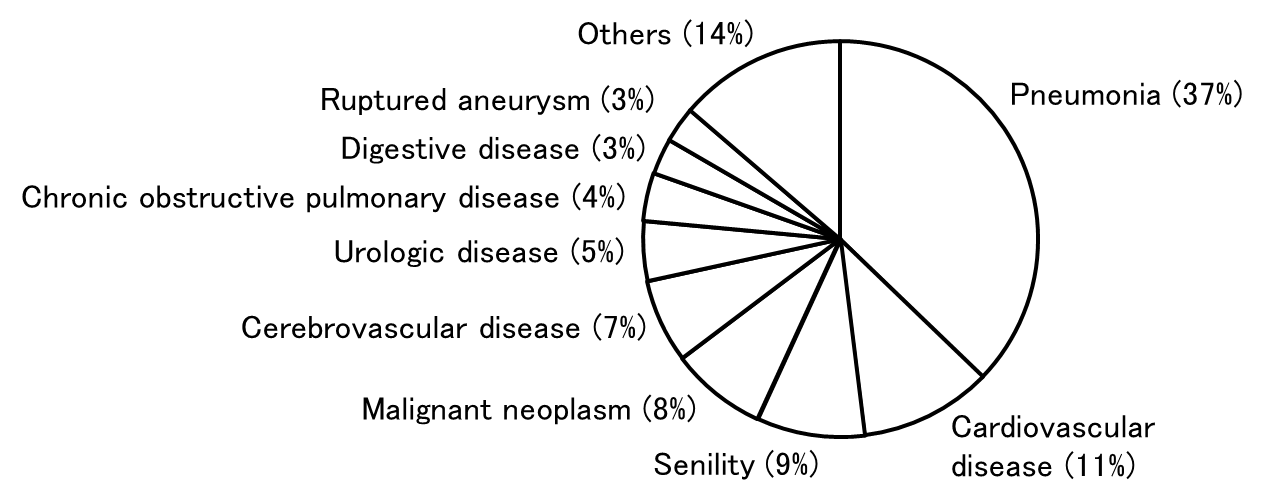


During the follow-up period, 102 residents died; pneumonia was identified as the most common cause of death (37% of deaths).

**Supplementary Tables**

Table S1: Independence degree of daily living for disabled older adults

| Rank J | Has some kind of disability but is almost independent in daily life and can go out on their own. |
| --- | --- |
| J1 | Uses public transportation or similar means to go out. |
| J2 | Goes out to nearby areas. |
| Rank A | Generally independent in indoor living, but does not go out without assistance. |
| A1 | Goes out with assistance and spends most of the day away from the bed. |
| A2 | Goes out infrequently and spends most of the day resting in bed. |
| Rank B | Requires some assistance for indoor living, and mostly spends the day in bed, but can maintain a seated position. |
| B1 | Transfers to a wheelchair and can eat and relieve oneself away from the bed. |
| B2 | Transfers to a wheelchair with assistance. |
| Rank C | Spends the entire day in bed, requiring assistance for elimination, meals, and dressing. |
| C1 | Can turn over in bed on their own. |
| C2 | Cannot turn over in bed on their own. |

Table S2: Independence degree of daily living for demented older adults

| Rank I | Has some form of dementia but is almost independent in daily life, both at home and socially. |
| --- | --- |
| Rank II | Despite some symptoms, behaviors, or communication difficulties that interfere with daily life, the individual can remain independent as long as someone is paying attention. |
| Rank IIa | The state described in Rank II is observed outside the home. |
| Rank IIb | The state described in Rank II is observed inside the home. |
| Rank III | There are symptoms, behaviors, or communication difficulties that interfere with daily life, and care is required. |
| Rank IIIa | The state described in Rank III is observed mainly during the day. |
| Rank IIIb | The state described in Rank III is observed mainly at night. |
| Rank IV | Frequent symptoms, behaviors, or communication difficulties that interfere with daily life are observed, and constant care is required. |
| Rank M | There are significant mental symptoms, problem behaviors, or serious physical conditions requiring specialized medical care. |

Table S3: Baseline characteristics of nursing home residents

|  |  |  |  |  | | n |
| --- | --- | --- | --- | --- | --- | --- |
| **Basic information** | | | |  |  |  |
|  | **Age, median (IQR), y** | | | 88.0 | (84.0–91.5) | 257 |
|  | **Sex, n (%)** | | |  |  |  |
|  |  | Male | | 55 | (21.4) |  |
|  |  | Female | | 202 | (78.6) |  |
|  | **BMI, median (IQR), kg/m^2^** | | | 19.7 | (17.9–21.5) | 256 |
|  |  |  | |  |  |  |
| **Comorbidities** | | |  |  |  |  |
|  | **HDS-R, median (IQR)** | | | 6.0 | (1.0–15.0) | 157 |
|  | **Charlson Comorbidity Index, median (IQR)** | | | 2.0 | (1.0–3.0) | 253 |
|  | **Cardiovascular disease, n (%)** | | |  |  |  |
|  |  | Yes | | 38 | (14.8) |  |
|  |  | No | | 219 | (85.2) |  |
|  | **Cerebrovascular disease, n (%)** | | |  |  |  |
|  |  | Yes | | 113 | (44.0) |  |
|  |  | No | | 144 | (56.0) |  |
|  | **Chronic lung disease, n (%)** | | |  |  |  |
|  |  | Yes | | 6 | (2.3) |  |
|  |  | No | | 251 | (97.7) |  |
|  | **Hepatic disease, n (%)** | | |  |  |  |
|  |  | Yes | | 16 | (6.3) |  |
|  |  | No | | 238 | (93.7) |  |
|  | **Diabetes mellitus, n (%)** | | |  |  |  |
|  |  | Yes | | 47 | (18.3) |  |
|  |  | No | | 210 | (81.7) |  |
|  | **Solid tumor, n (%)** | | |  |  |  |
|  |  | Yes | | 27 | (10.5) |  |
|  |  | No | | 230 | (89.5) |  |
|  |  |  | |  |  |  |
| **Vaccinations** | | |  |  |  |  |
|  | **Pneumococcal vaccine, n (%)** | | |  |  |  |
|  |  | Yes | | 105 | (49.5) |  |
|  |  | No | | 107 | (50.5) |  |
|  | **Influenza vaccine, n (%)** | | |  |  |  |
|  |  | Yes | | 212 | (98.6) |  |
|  |  | No | | 3 | (1.4) |  |
|  |  |  | |  |  |  |
| **Medications** | | |  |  |  |  |
|  | **ACE inhibitors, n (%)** | | |  |  |  |
|  |  | Yes | | 10 | (3.9) |  |
|  |  | No | | 247 | (96.1) |  |
|  | **Gastric acid-suppressive drugs, n (%)** | | | |  |  |
|  |  | Proton pump inhibitor | | 67 | (26.1) |  |
|  |  | Histamine H2-receptor antagonists | | 79 | (30.7) |  |
|  |  | No | | 111 | (43.2) |  |
|  | **Anticholinergic agents, n (%)** | | |  |  |  |
|  |  | Yes | | 7 | (2.7) |  |
|  |  | No | | 250 | (97.3) |  |
|  | **Steroids, n (%)** | | |  |  |  |
|  |  | Yes | | 8 | (3.1) |  |
|  |  | No | | 249 | (96.9) |  |
|  | **Hypnotics, n (%)** | | |  |  |  |
|  |  | Yes | | 66 | (25.7) |  |
|  |  | No | | 191 | (74.3) |  |
|  | **Antipsychotics, n (%)** | | |  |  |  |
|  |  | Yes | | 72 | (28.0) |  |
|  |  | No | | 185 | (72.0) |  |
|  |  |  | |  |  |  |
| **Laboratory data** | | |  |  |  |  |
|  | **Serum hemoglobin, median (IQR), g/dL** | | | 11.6 | (10.5–12.9) | 252 |
|  | **Serum albumin, median (IQR), g/dL** | | | 3.6 | (3.4–3.9) | 247 |
|  | **Serum total protein, median (IQR), g/dL** | | | 6.6 | (6.3–7.0) | 244 |
|  | **Serum total cholesterol, median (IQR), mg/dL** | | | 170.0 | (147.0–196.0) | 240 |
|  | **eGFR, median (IQR), mL/min/1.73 m^2^** | | | 62.5 | (48.2–80.0) | 253 |
|  |  |  |  |  |  |  |
| **Information in the doctor’s written opinion for long-term care insurance** | | | | | |  |
|  | **Nursing care level, n (%)** | | | |  |  |
|  |  | Support Required 1 (High ADL) | | 0 | (0,0) |  |
|  |  | Support Required 2 | | 0 | (0,0) |  |
|  |  | Care Level 1 | | 9 | (5.9) |  |
|  |  | Care Level 2 | | 7 | (4.6) |  |
|  |  | Care Level 3 | | 32 | (21.1) |  |
|  |  | Care Level 4 | | 74 | (48.7) |  |
|  |  | Care Level 5 (Low ADL) | | 30 | (19.7) |  |
|  | **Independence degree of daily living for disabled older adult, n (%)** *^1^ | | | | | |
|  |  | independent | | 0 | (0.0) |  |
|  |  | J1 (High ADL) | | 0 | (0.0) |  |
|  |  | J2 | | 2 | (0.8) |  |
|  |  | A1 | | 13 | (5.1) |  |
|  |  | A2 | | 31 | (12.1) |  |
|  |  | B1 | | 38 | (14.8) |  |
|  |  | B2 | | 118 | (45.9) |  |
|  |  | C1 | | 37 | (14.4) |  |
|  |  | C2 (Low ADL) | | 18 | (7.0) |  |
|  | **Independence degree of daily living for demented older adult, n (%)** *^2^ | | | | | |
|  |  | independent | | 7 | (2.7) |  |
|  |  | I (High ADL) | | 18 | (7.0) |  |
|  |  | IIa | | 18 | (7.0) |  |
|  |  | IIb | | 37 | (14.4) |  |
|  |  | IIIa | | 111 | (43.2) |  |
|  |  | IIIb | | 31 | (12.1) |  |
|  |  | IV | | 32 | (12.5) |  |
|  |  | M (Low ADL) | | 3 | (1.2) |  |
|  | **Impairment of short-term memory, n (%)** | | | | |  |
|  |  | Yes | | 230 | (90.2) |  |
|  |  | No | | 25 | (9.8) |  |
|  | **Cognitive ability for daily decision-making, n (%)** | | | | |  |
|  |  | independent | | 17 | (6.6) |  |
|  |  | semi-independent | | 59 | (23.0) |  |
|  |  | needing help | | 93 | (36.3) |  |
|  |  | impossible | | 87 | (34.0) |  |
|  | **Ability to communicate intentions, n (%)** | | | | |  |
|  |  | possible | | 36 | (14.1) |  |
|  |  | a little difficult | | 60 | (23.5) |  |
|  |  | only specific requests | | 103 | (40.4) |  |
|  |  | impossible | | 56 | (22.0) |  |
|  | **Weight change over the past 6 months (≥3% change), n (%)** | | | | |  |
|  |  | increase or stable | | 172 | (83.5) |  |
|  |  | decrease | | 34 | (16.5) |  |
|  | **Eating ability, n (%)** | | | | |  |
|  |  | independent | | 192 | (75.0) |  |
|  |  | complete assistance | | 64 | (25.0) |  |
|  | **Social interaction, n (%)** | | | | |  |
|  |  | independent | | 34 | (13.3) |  |
|  |  | partial assistance | | 140 | (54.9) |  |
|  |  | complete assistance | | 81 | (31.8) |  |

Abbreviations:

ACE inhibitor, angiotensin converting enzyme inhibitor; ADL, activity of daily living; BMI, body mass index; eGFR, estimated glomerular filtration rate; HDS-R, Hasegawa dementia rating scale-revised; IQR, interquartile range

*^1^ Details of independence degree of daily living for disabled older adults are shown in Table S1.

*^2^ Details of independence degree of daily living for demented older adults are shown in Table S2.
